# Gap Effect Abnormalities during a Visually Guided Pro-Saccade Task in Children with Attention Deficit Hyperactivity Disorder

**DOI:** 10.1371/journal.pone.0125573

**Published:** 2015-05-27

**Authors:** Yuka Matsuo, Masayuki Watanabe, Masako Taniike, Ikuko Mohri, Syoji Kobashi, Masaya Tachibana, Yasushi Kobayashi, Yuri Kitamura

**Affiliations:** 1 Department of Social and Environmental Medicine, Graduate School of Medicine, Osaka University, Suita, Osaka, Japan; 2 Department of Biomedical and Molecular Sciences, Queens University, Kingston, Ontario, Canada; 3 Molecular Research Center for Children’s Mental Development, United Graduate School of Child Development, Osaka University, Suita, Osaka, Japan; 4 Department of Electrical Engineering and Computer Sciences, Graduate School of Engineering, University of Hyogo, Shosha, Himeji, Hyogo, Japan; 5 Department of Neuroscience, Mayo Clinic Florida, Jacksonville, Florida, United States of America; 6 Visual Neuroscience Laboratory, Graduate School of Frontier Biosciences, Osaka University, Suita, Osaka, Japan; 7 Center for Information and Neural Networks, National Institute of Information and Communications Technology and Osaka University, Suita, Osaka, Japan; University of Reading, UNITED KINGDOM

## Abstract

Attention deficit hyperactivity disorder (ADHD) is a neurodevelopmental disorder that starts in early childhood and has a comprehensive impact on psychosocial activity and education as well as general health across the lifespan. Despite its prevalence, the current diagnostic criteria for ADHD are debated. Saccadic eye movements are easy to quantify and may be a quantitative biomarker for a wide variety of neurological and psychiatric disorders, including ADHD. The goal of this study was to examine whether children with ADHD exhibit abnormalities during a visually guided pro-saccadic eye-movement and to clarify the neurophysiological mechanisms associated with their behavioral impairments. Thirty-seven children with ADHD (aged 5–11 years) and 88 typically developing (TD) children (aged 5–11 years) were asked to perform a simple saccadic eye-movement task in which step and gap conditions were randomly interleaved. We evaluated the gap effect, which is the difference in the reaction time between the two conditions. Children with ADHD had a significantly longer reaction time than TD children (p < 0.01) and the gap effect was markedly attenuated (p < 0.01). These results suggest that the measurement of saccadic eye movements may provide a novel method for evaluating the behavioral symptoms and clinical features of ADHD, and that the gap effect is a potential biomarker for the diagnosis of ADHD in early childhood.

## Introduction

Attention deficit hyperactivity disorder (ADHD) is one of the most common developmental disorders and is characterized by behavioral and cognitive symptoms such as inattention, impulsiveness, and hyperactivity [1–8]. Although studies on the pathophysiology of ADHD, including neuroimaging and pharmacological studies, are accumulating [3, 9–21], the clinical diagnosis and treatment of this disorder still rely on subjective evaluation of the behavioral symptoms using questionnaires answered by parents and teachers. To improve clinical practice, it is important to develop objective methods to evaluate the behavioral symptoms of the disorder based on evidence derived from experimental studies.

There are relatively few studies of saccadic eye movements in children with ADHD [22, 23]. However, eye movements have been studied extensively as a potential quantitative biomarker for a wide variety of neurological and psychiatric disorders, including ADHD [24–28], and are particularly attractive as a potential quantitative biomarker for ADHD for two reasons: (a) the neural circuits that control eye movements have been well-established in neurophysiological studies in animals [21, 29, 30] and in neuroimaging studies in humans [11, 13, 31–35], and (b) there are overlaps between the brain structures that control eye movements and those suggested to be dysfunctional in ADHD, i.e., the prefrontal cortices and the basal ganglia. Therefore, the analysis of eye movements in ADHD may provide a unique and objective method to diagnose the disorder and address its etiology.

A fundamental oculomotor behavior involves of a series of alternations between fixation and saccade guided by visual information, and this behavior occurs hundreds of thousands of times a day. Children with ADHD have problems with visual fixation and also exhibit several deficits in visually guided saccades, including a longer time to initiate saccades in response to the appearance of the target [27, 36–43]. In adult ADHD, several studies showed abnormal preparatory states preceding a saccade response [44–45]. In this study, we examined the gap effect, in which saccadic initiation is facilitated by the disappearance of the central fixation point, with simultaneous or delayed target appearance [20, 46–49]. The gap effect is thought to be due to reflexive and volitional processing of visual stimuli in the superior colliculus, a midbrain structure that controls both fixation maintenance and saccade initiation [50]. A recent neurophysiological study in behaving monkeys showed that neural mechanisms of volitional processing are involved in the gap effect as well as reflexive processing [51].

A wide variety of paradigms that challenge cognitive functions such as go/no-go behavioral inhibition [25, 27, 28, 37–40] and working memory [25, 27, 28] have been used to characterize saccades in children with ADHD. By contrast, in this study, we adopted a very simple paradigm in which children were asked to follow visual stimuli using saccadic eye movements. We selected this paradigm because patients with ADHD exhibit eye-movement abnormalities in this task, but patients with other developmental disorders do not [28]. Furthermore, the simple instructions required for this paradigm are very easy to understand and can be understood by children of a young age [28, 41].

We found that the gap effect was attenuated in children with ADHD compared with that in typically developing (TD) children. This is partly consistent with previous reports [36–39] showing that children with ADHD have a longer reaction time than those with TD [39] and that reaction time decreases with age [25, 52–53]. We hypothesize that there are two types of signals involved in the mechanism of disengagement from an initial fixation point, reflexive and volitional signals, and propose a conceptual model of the mechanism underlying the difference in the gap effect between ADHD and TD controls.

## Materials and Methods

### Participants

Thirty-seven children with ADHD (12 female and 25 male; age range, 5–11 years; mean age ± standard deviation, 7.9 ± 1.7 years) recruited from the Molecular Research Center for Children’s Mental Development at Osaka University Hospital and 88 TD children (35 female and 53 male; age range, 5–11 years; mean age ± standard deviation, 7.8 ± 1.9 years) without any visual, neurological, or psychiatric disorders participated in the study. The study was approved by the Internal Review Board of Osaka University Medical School Hospital, Suita, Japan (approval code 09088). All participants and their parents provided written informed consent prior to participating.

All ADHD children underwent a semi-structured diagnostic interview for developmental disorders and a video-based behavioral investigation performed by senior expert pediatricians. The ADHD Rating Scale (ADHD-RS; 4^th^ edition, Home Version), which is based on the Diagnostic and Statistical Manual of Mental Disorders, 4^th^ edition (DSM-IV), criteria for ADHD and is considered to be effective for diagnosis of this disorder [54–56], consists of nine inattention and nine hyperactivity and impulsivity criteria, all rated on a four-point scale (0 = never [less than once a week], 1 = sometimes [several times a week], 2 = often [once a day], and 3 = very often [several times a day]). Three measures were taken from the ADHD-RS: hyperactivity and impulsivity score (range, 0–27), inattention score (range, 0–27), and total score (range, 0–54) over the previous 6 months as rated by the parents of the children with ADHD. The Child Behavior Checklist (CBCL), which is a common behavior-rating scale used to assess emotional, behavioral and social aspects of life in children and adolescents [57–58], consists of 113 questions all rated on a three-point scale (0 = absent, 1 = occurs sometimes, 2 = occurs often). The time frame for item responses is the past six months and two versions of the checklist, i.e., the preschool version (CBCL/1^1/2^-5) for children aged 18 months to 5 years and the school-age version (CBCL/6-18) for children aged 6 to 18 years, are available. The CBCL score can be subdivided into scores for eight symptoms: withdrawn, somatic complaints, anxious/depressed, social problems, thought problems, attention problems, delinquent behavior and aggressive behavior. We obtained CBCL profiles for children with ADHD and TD controls. Expert pediatricians yielded a definitive diagnosis of ADHD based on the DSM-IV criteria.

### Visually guided saccade paradigm

For the visually guided saccade paradigm, a central fixation point (diameter, 10.7 mm) appeared for 700 ms on a cathode-ray tube display placed 30 cm in front of the subject. This was followed by the appearance of a peripheral stimulus (diameter, 10.7 mm) to the left or right of the central fixation point at a visual angle of 16.4° for 700 ms. Children were instructed to initially maintain their eyes on the fixation point and to move their eyes toward the peripheral stimulus in response to its appearance as quickly and accurately as possible. The instructions were easily understood by participants regardless of their age and the severity of their clinical condition.

The saccade paradigm was performed in two conditions that were randomly interleaved in each block of trials. In the step condition, the peripheral stimulus appeared at the same time as the fixation point disappeared. In the gap condition, the peripheral stimulus appeared 200 ms after the fixation point disappeared. Each participant completed three blocks of 40 trials, with a 30 s rest between consecutive blocks.

### Data collection and analysis

Horizontal eye movements were detected using an infrared limbus detection eye-tracking device (T.K.K.2930a; Takei, Japan). The signal was sampled at 1 kHz and low-pass filtered at 100 Hz. Experiment Builder (SR Research Ltd., Germany) was used to generate the visual stimuli, and LabView (National Instruments Ltd., USA) was used to determine the exact time at which a visual stimulus appeared and disappeared (using the transistor-transistor logic compatible signal) and to record continuous eye positions as digitally converted signals. Off-line analysis was performed using Matlab (MathWorks, USA) software.

The onset and end of each saccade were identified using radial eye velocity criteria (threshold, 30°/s). Detected saccades were analyzed if they satisfied the following criteria: amplitude >2°, peak velocity >50°/s, and duration >20 ms. Saccadic reaction time was defined as the time from the appearance of the peripheral stimulus to the initiation of the saccade. We also used an exclusion criteria for anticipatory saccade: reaction time <70 ms [59]. Saccades were scored as correct if the first movement after the appearance of the peripheral stimulus was in the correct direction.

### Statistical analysis

A three-way analysis of variance (ANOVA) was used to compare the saccade reaction time across saccade conditions (gap *vs*. step; within-subject factor), subject groups (TD *vs*. ADHD; between-subject factor), and age groups (seven levels: 5 years, 6 years, 7 years, 8 years, 9 years, 10 years and 11 years; between-subject factor). The gap effect was quantified for each subject by subtracting the average reaction time for the gap condition from the average reaction time for the step condition. A two-way ANOVA was used to compare the gap effect across subject groups (TD *vs*. ADHD; between-subject factor) and age groups (seven levels; between-subject factor). The variability in the reaction time was quantified for each subject using the coefficient of variation. A three-way ANOVA was used to compare the coefficient of variation across saccade conditions (gap *vs*. step; within-subject factor), subject groups (TD vs. ADHD; between-subject factor), and age groups (seven levels; between-subject factor).

The gap effect was also evaluated using a receiver operating characteristic (ROC) analysis. The ROC value is the area under the ROC curve (auROC), and auROC >0.5 means that the reaction time for step trials was longer than for gap trials and indicates a different distribution of the reaction time in the gap and the step trials. A two-way ANOVA was used to compare auROC across subject groups (TD *vs*. ADHD; between-subject factor) and age groups (seven levels; between-subject factor). To examine the relationship between the gap effect and the behavioral symptoms of ADHD, the relationship between auROC and ADHD-RS scores with a fixing age analysis was evaluated using Spearman’s partial correlation coefficient.

## Results

### Participant characteristics

**Fig 1:**
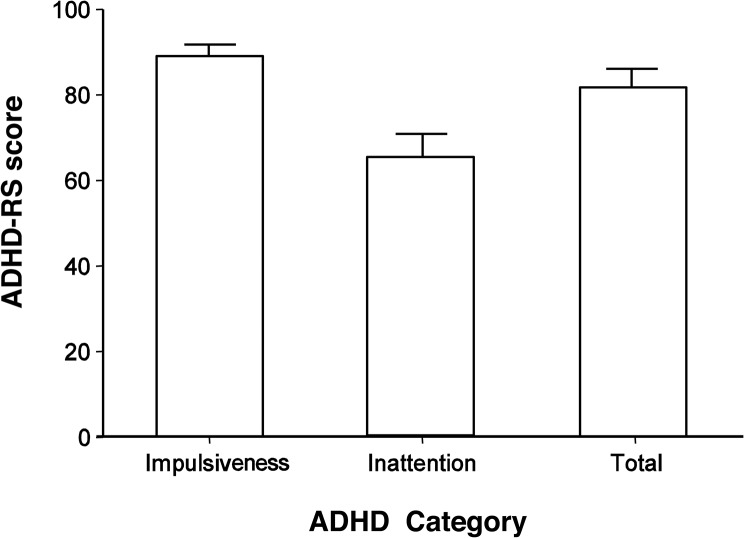
Attention Deficit Hyperactivity Disorder Rating Scale (ADHD-RS) score in ADHD children. The bars indicate the average score on the impulsivity/hyperactivity section of the ADHD-RS, the inattention section of the ADHD-RS, and the total ADHD-RS score for all 37 children with ADHD. Error bars denote the standard deviation.

**Fig 2:**
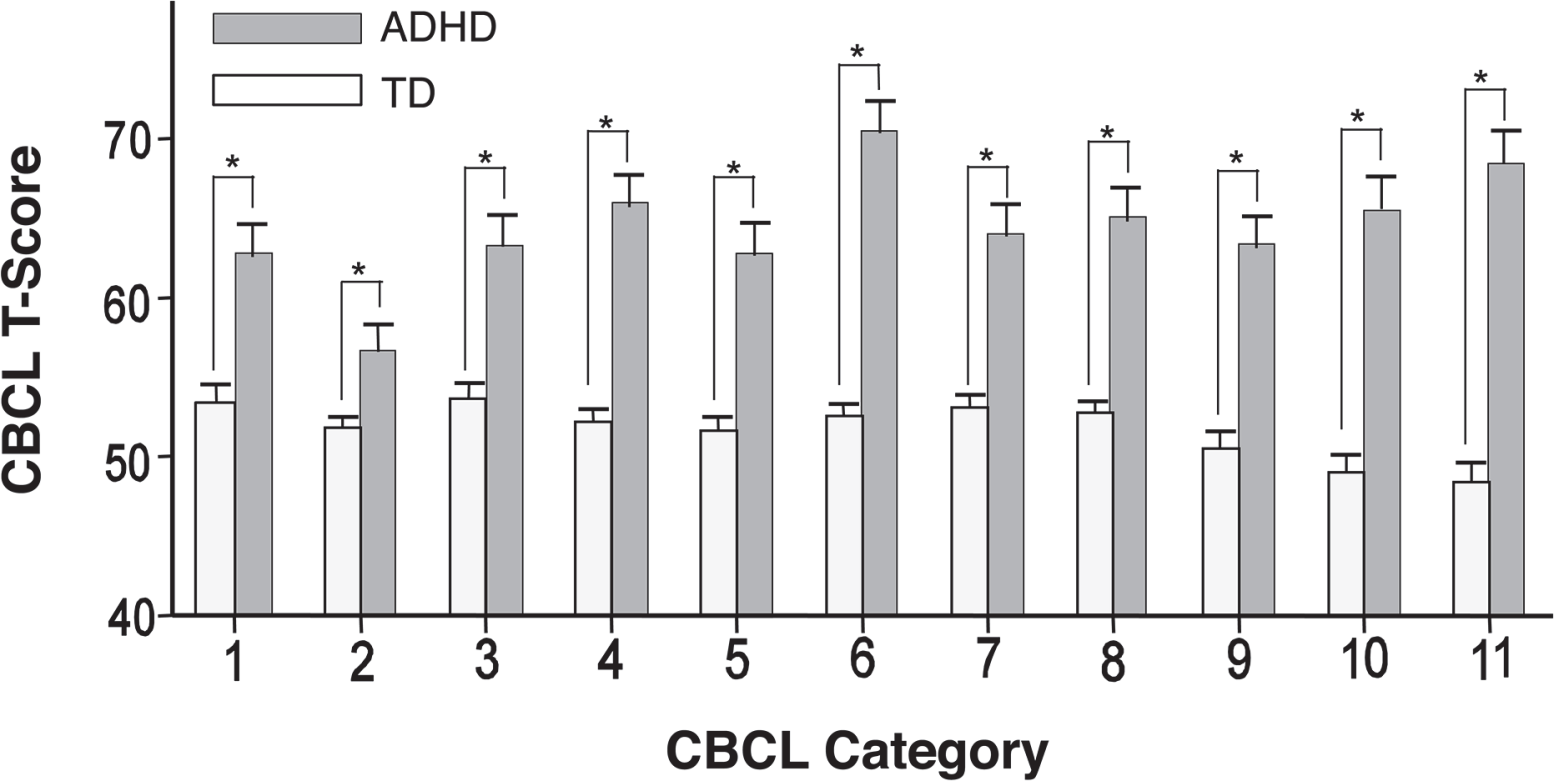
Score for each symptom assessed by the Child Behavior Check List (CBCL). The patterned gray and white bars indicate the average T-score in children with attention deficit hyperactivity disorder (ADHD; n = 37) and typically developing (TD) children (n = 88), respectively. Numbers on the x-axis denote the CBCL subcategories: 1 = Withdrawn; 2 = Somatic complaints; 3 = Anxious/depressed; 4 = Social problems; 5 = Thought problems; 6 = Attention problems; 7 = Delinquent behavior; 8 = Aggressive behavior; 9 = Internalizing problems; 10 = Externalizing problems; 11 = Total score across all categories. Error bars represent the standard deviation. Asterisks (*) indicate statistically significant differences between the two groups (p < 0.01).

The average score for the hyperactivity/impulsivity and inattention subscales of the ADHD-RS and the average total score for the ADHD-RS are shown in Fig 1. The T-score of the ADHD children and TD children for each symptoms evaluated by the CBCL is shown in Fig 2. Statistically significant differences were observed between the two groups for all CBCL symptoms. In the TD group, 21 of the 88 children (23.9%) exhibited more than one CBCL symptoms score that was beyond the standardized cut-off point for Japanese children; however, they were not excluded from the TD group because the results did not vary significantly according to their inclusion or exclusion (data not shown).

### Saccade reaction time and gap effect

**Fig 3:**
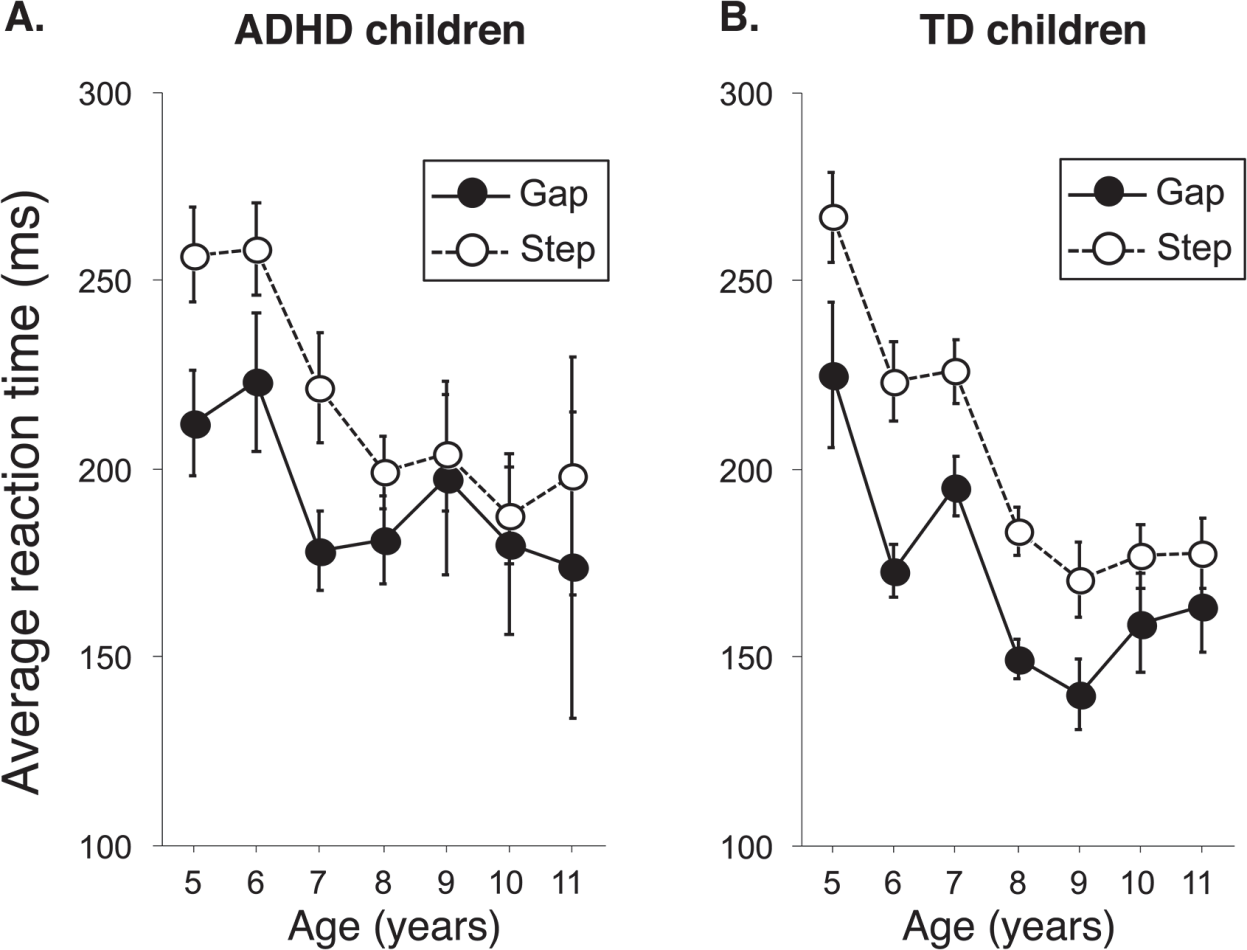
Saccade reaction time during gap and step trials. Data are shown for children with attention deficit hyperactivity disorder (ADHD) (A) and typically developing (TD) children (B). Black circles with continuous lines represent gap trials and white circles with broken lines represent step trials. Error bars denote the standard deviation.

ANOVA revealed significant main effects of saccade condition (F_(1)_ = 149.66, p < 0.0001), subject group (F_(1)_ = 5.25, p < 0.05), and age group (F_(6)_ = 11.07, p < 0.0001) on saccade reaction time and a significant two-way interaction between age group and saccade condition (F_(6)_ = 3.09, p < 0.01). None of the other interactions were significant (p > 0.05). In both the gap and the step conditions, saccade reaction time decreased with age (Fig 3). Visual inspection of the data shows that the difference between the gap and step conditions was larger in the TD group than in the ADHD group (Fig 3).

The gap effect was calculated as the difference in the average reaction time in the two conditions. ANOVA revealed a significant main effect of age group (F_(6)_ = 2.51, p < 0.05) on gap effect but no effect of subject group (F_(1)_ = 0.92, p > 0.3) and no interaction (F_(6)_ = 0.99, p > 0.4). ANOVA also revealed a significant main effect of age group (F_(6)_ = 5.21, p < 0.0005) on the coefficient of variation for reaction time and a significant two-way interaction between age group and subject group (F_(6)_ = 2.19, p < 0.05). However, it is difficult to interpret the difference in the gap effect calculated as the difference in the average reaction time of the two conditions because the variance of reaction time is influenced by age and subject group, as shown in previous studies [28, 38, 39].

**Fig 4:**
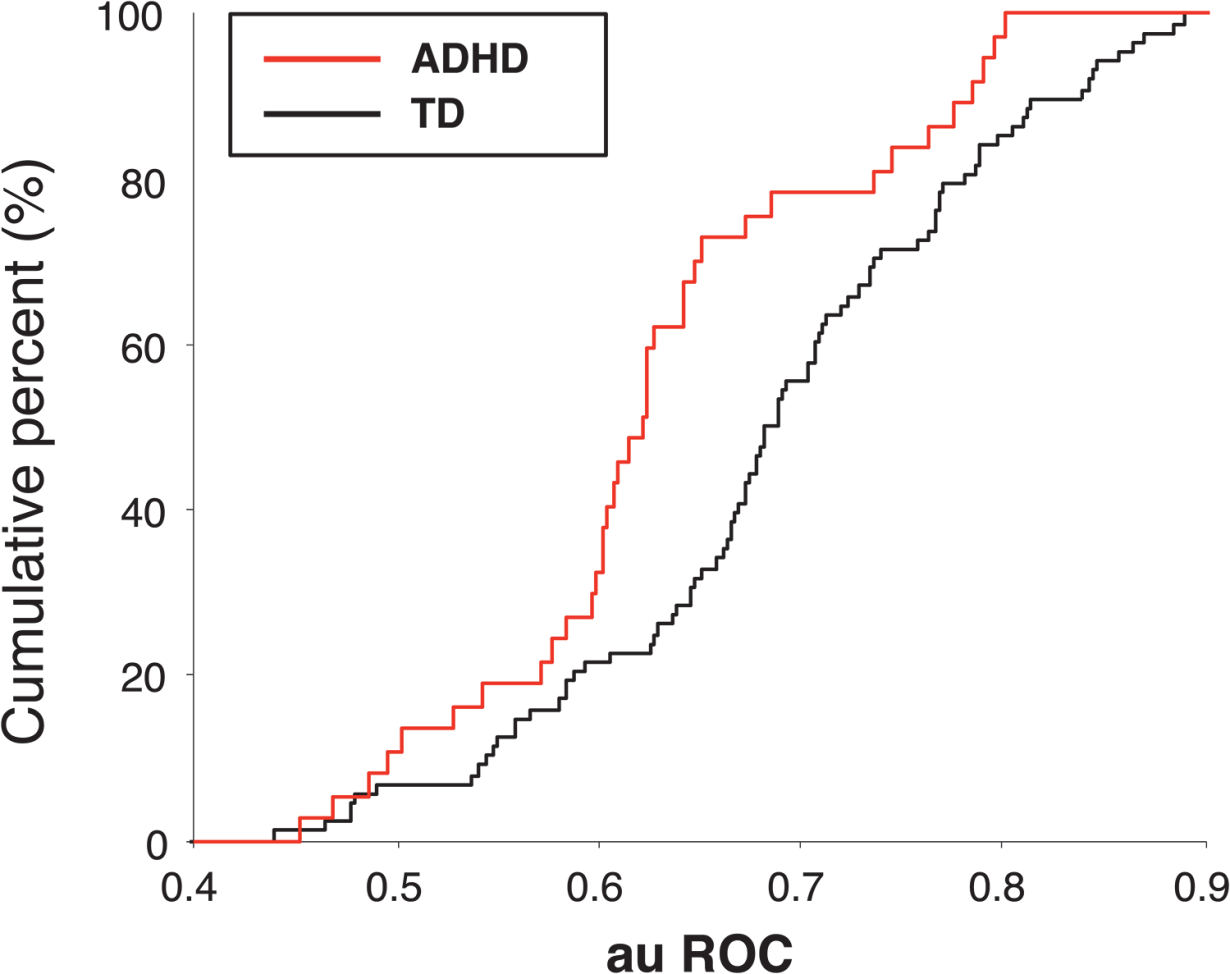
Cumulative distribution of the area under the receiver operating characteristic curve (auROC). The red line shows the cumulative percentage for children with attention deficit hyperactivity disorder (ADHD), and the black line shows the cumulative percentage for typically developing (TD) children. An auROC > 0.5 indicates a shorter reaction time in gap trials than in step trials, i.e., the existence of a gap effect. A Wilcoxon rank-sum test revealed that the distribution of the auROC was significantly smaller for ADHD than for TD (p = 0.00281).

ROC analysis does not depend on the variance of reaction times and instead evaluates the distribution of the reaction times. Fig 4 summarizes the auROC for both groups. The auROC was >0.5 in both the TD group (one-sample *t* test: *t*_(87)_ = 16.6, p < 0.0001) and the ADHD group (*t*_(36)_ = 8.35, p < 0.0001), confirming the presence of a gap effect in both groups. However, the auROC was smaller in the ADHD group than in the TD group (two-sample *t* test: *t*_(123)_ = 2.82, p < 0.01). ANOVA revealed a significant main effect of subject group on auROC (F_(1)_ = 5.87, p < 0.05) but no significant effect of age group (F_(6)_ = 0.4, p > 0.8) and no interaction (F_(6)_ = 1.38, p > 0.2). These results demonstrate that the gap effect quantified using ROC analysis was attenuated in the ADHD group compared with the TD group, regardless of age.

### Correlation between auROC and clinical features

There was no significant correlation between the auROC and the ADHD-RS score (|r| < 0.14, p > 0.4) or between the auROC and the T-score for any CBCL symptoms (|r| < 0.22, p > 0.2) in ADHD children (Table 1). Similarly, there was no significant correlation between auROC and the T-score for any CBCL symptoms in TD children (|r| < 0.21, p > 0.05). There were almost no significant correlations between the T-score for each CBCL symptom and reaction time, its coefficient of variance, and the ratio of correct to incorrect responses in the gap and step conditions in the ADHD and TD groups. Only the T-score for the social problems symptom on the CBCL showed a significant correlation with the coefficient of variance in the gap condition and the ratio of correct-to-incorrect responses in the step condition (Table 1).

**Table 1:** Correlation coefficients between eye-movement parameters and ADHD rating scales (ADHD-RS), and child behavior checklist (CBCL) subcategories in ADHD and typically developing (TD) groups.

	Gap Effect	SRT (m)		C. V.		Correct Responses	
	(auROC)	gap	step	gap	step	gap	step
**ADHD-rating scale**							
Hyperactivity/Impulsiveness	−0.10	−0.01	−0.08	−0.09	0.01	0.22	−0.04
Inattention	−0.14	0.02	−0.01	0.05	0.06	0.18	0.18
Total	−0.08	−0.13	−0.20	−0.20	−0.05	0.26	0.15
**CBCL subcategories**							
**ADHD group**							
Withdrawn	0.11	−0.15	−0.01	−0.25	−0.08	0.09	0.14
Somatic Complaints	0.05	0.06	0.11	−0.14	0.09	0.16	−0.07
Anxious/Depressed	0.22	−0.26	−0.15	−0.23	−0.06	0.2	0.29
Social Problems	0.08	−0.26	−0.21	−0.42	−0.16	0.31	0.37
Thought Problems	−0.06	−0.13	−0.21	−0.24	−0.22	0.08	0.09
Attention Problems	−0.05	−0.04	−0.09	−0.28	−0.22	0.19	0.23
Delinquent Behavior	−0.04	−0.13	−0.14	−0.19	−0.18	0.26	0.31
Aggressive Behavior	−0.01	−0.11	−0.19	−0.27	−0.18	0.23	0.26
Internalizing Problems	0.18	0.04	−0.10	−0.28	−0.09	0.19	0.24
Externalizing Problems	−0.02	0.15	−0.18	−0.29	−0.18	0.27	0.29
Total Problems	0.07	0.16	−0.17	−0.38*	−0.19	0.23	0.28
**TD group**							
Withdrawn	−0.11	0.04	0.01	0.11	0.18	0.03	−0.10
Somatic Complaints	0.03	0.01	0.04	−0.05	−0.16	0.04	0.03
Anxious/Depressed	−0.16	0.08	0.03	−0.07	0.04	0.17	0.11
Social Problems	−0.06	0.02	−0.06	−0.04	−0.05	0.05	0.18
Thought Problems	−0.14	0.02	−0.06	0.16	−0.02	0.11	0.16
Attention Problems	−0.15	0.06	−0.02	0.08	0.05	0.09	0.04
Delinquent Behavior	−0.09	0.13	0.07	0.05	−0.14	0.14	0.23
Aggressive Behavior	−0.20	0.22	0.16	0.14	−0.08	0.15	0.14
Internalizing Problems	−0.11	0.04	0.02	−0.08	0.05	0.15	0.1
Externalizing Problems	−0.11	0.15	0.13	0.13	−0.12	0.08	0.16
Total Problems	−0.16	0.16	0.11	0.04	−0.03	0.12	0.1

Values are correlation coeffeicients from an exact test in the Spearman's partial correlation coefficients with age fixed

* denotes a significantly different at p < 0.05. SRT(m) = Mean value of the saccadic reaction times. C.V. = Coefficient of variance of reaction times. auROC = Area under the receiver operationg characteristic curve.

## Discussion

We found that the gap effect was attenuated in children with ADHD compared with that in TD controls, regardless of age. The gap effect did not correlate with ADHD-RS or CBCL scores, suggesting that the gap effect may capture deficits that are not identified by these qualitative behavioral evaluations. In the following sections, we discuss the estimation of the gap effect, the mechanism underlying the attenuation of the gap effect in ADHD, and the application of this measure to clinical practice in the future.

The gap effect is normally quantified by subtracting the average reaction time in the gap condition from that in the step condition. This estimate decreases with age [53] and does not differentiate between children with ADHD and TD controls [39], which is consistent with our findings. However, the gap effect calculated using this method might be underestimated, as reaction times are variable and decrease with age. Our results confirmed that the coefficient of variance of reaction time decreased with age and was influenced by subject group (ADHD *vs*. TD). Therefore, we used ROC analyses to evaluate the difference in saccade reaction time between gap and step trials. In ROC analysis, the area under the curve represents the power of discrimination between gap and step trials, as shown in previous studies [37–38].

We adopted a gap-step task instead of another task to keep the behavioral paradigm as simple as possible. In our paradigm, the disappearance of the fixation point always indicated the appearance (in the step condition) or upcoming appearance (in the gap condition) of a target. Thus, we speculate that the disappearance of the central fixation point facilitated saccade initiation in all trials. By contrast, in behavioral paradigms that use overlap and gap conditions, the disappearance of the central fixation point can facilitate saccade initiation in only half of the trials.

**Fig 5:**
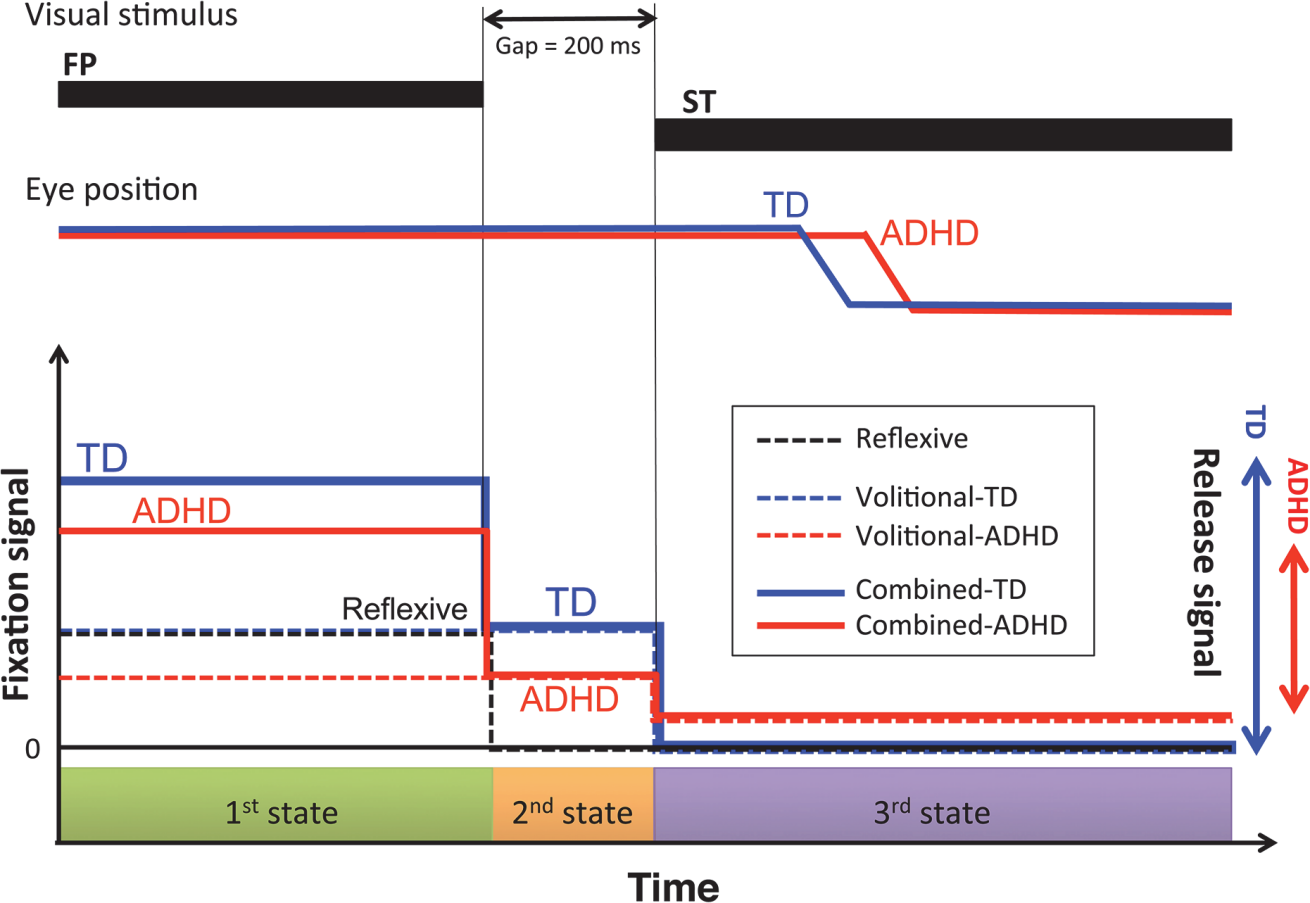
Schematic representation of fixation control during a gap trial. **Top panel:** FP and ST denote the fixation point and saccade target, respectively. The blue and red lines denote eye movements for typically developing (TD) children and children with attention deficit hyperactivity disorder (ADHD), respectively. **Bottom panel:** We hypothesize that two types of signal influence the maintenance of eye-movement to fixate: reflexive and volitional. The dashed black line represents the magnitude of the reflexive signal, which depends solely on presence or absence of the fixation point. The dashed blue and red lines represent the magnitude of volitional signals in TD and ADHD, respectively. The combination of the reflexive and volitional fixation signals yields the three sequential states depicted here: (1) maintenance of fixation until the fixation point disappears (reflexive and volitional fixation signals both exist), (2) disengagement from the fixation point and preparation for initiation of a saccade (reflexive fixation signal absent; volitional fixation signals exist) and (3) triggering of saccade as the stimulus target appears (reflexive and volitional fixation signals both minimal). Solid blue and red lines represent the total magnitude of the reflexive and volitional signals in of the each three states, i.e., the magnitude of total fixation command. The difference in the magnitude of the total fixation command between state 1 and state 3 reflects the release signal, depicted by arrows on the right side of the figure.

Fig 5 shows a conceptual model of the mechanisms underlying the attenuated gap effect in children with ADHD. It has been hypothesized that two types of signals control the reaction time of visually guided pro-saccadic eye movements: reflexive signals and volitional signals [40, 51]. Watanabe et al. suggested that the integration of reflexive and volitional signals to engage gaze onto an initial fixation point presented in the central field is required to account for the gap effect during a visually guided saccade [50], and we have extended this model to explain the gap effect abnormalities in ADHD. The reflexive component of the fixation signal (dashed black line in the bottom panel of Fig 5) depends solely on the presence or absence of the initial fixation point, and disappears immediately after the central fixation point disappears. By contrast, volitional components (dashed blue and red lines in the bottom panel of Fig 5) are maintained until the target stimulus appears in the peripheral field (see the top panel of Fig 5) because children are instructed to track the target stimulus and suppress anticipatory or accidental eye movements. Volitional signals are influenced by additional factors, such as the gap duration (200 ms in this study) and the duration of the presentation of the fixation point (700 ms in this study), which can be used to predict the timing of stimulus appearance and prepare for saccade initiation. We have assumed that the reflexive and volitional signals are additive. Because of the difference in temporal dynamics between the reflexive and volitional signals, the combination of these two signals must yield the following three sequential states: (1) maintenance of engagement onto the central fixation point (reflexive and volitional components both present), (2) disengagement from the central fixation point and preparation to initiate saccade (reflexive component absent; volitional component present) and (3) triggering of saccade as a target stimulus appears (reflexive and volitional components both minimal). The total magnitude of the reflexive and volitional signals in each of the three states dictates the total amount of fixation signal (solid blue and red lines in the bottom panel of Fig 5). Consequently, the difference in the fixation signal between state 1 and state 3 corresponds to the release signal that is necessary to provide relevant reaction time in the visually guided pro-saccade task. To account for the marked attenuation of the gap effect in children with ADHD, we hypothesize that the volitional signal is affected by any disturbance of signal transmission through the three states, whereas the reflexive signal is intact and similar to that in TD children. As the volitional signal is affected, the total magnitude of the reflexive and volitional signals in states 1 and 2 is lower in children with ADHD than in TD children. In state 3, the affected volitional signal causes inadequate reduction in the total amount of fixation signal and, consequently, results in attenuation of the release signal, i.e., the difference in the total fixation signal between state 1 and state 3. Our model can also explain the longer reaction time and less stable eye-movement during the fixation period in children with ADHD [25, 27, 28, 36–40].

We designed this model to provide the simplest possible explanation for the saccade abnormalities observed in ADHD. Recently Watanabe et al. suggested that fixation saccades alter the gap effect, presumably by disrupting volitional saccade preparation [60]. However, an important limitation of this model is that it does not take into account execution signals for saccades, which may also be dysfunctional in ADHD. Because fixation signals and execution signals interact before saccade initiation, it is difficult to disentangle these two factors based on the analyses of saccades performed to date. Future research should combine the analysis of saccades with neuroimaging techniques to further develop our model.

We did not find significant correlations or trends between the gap effect or other eye-movement parameters and clinical features, except for the social problems domain of the CBCL (Table 1). This may indicate that the gap effect reflects biological features rather than standard measures such as the ADHD-RS and CBCL, which are currently used for clinical assessment. As in Huntington’s and Parkinson’s disease [61–62], the attenuated gap effect might reflect skeletomotor coordination deficits, which have been reported in ADHD [63].

In future studies we aim to assess the utility of eye-movement measures as a specific biomarker for the diagnosis of ADHD in early childhood and to evaluate the neurodevelopmental state related to cognitive and executive functions observed via social behaviors.
